# Bar-HRM for Authentication of Plant-Based Medicines: Evaluation of Three Medicinal Products Derived from Acanthaceae Species

**DOI:** 10.1371/journal.pone.0128476

**Published:** 2015-05-26

**Authors:** Maslin Osathanunkul, Panagiotis Madesis, Hugo de Boer

**Affiliations:** 1 Department of Biology, Faculty of Science, Chiang Mai University, Chiang Mai, Thailand; 2 Institute of Applied Biosciences, Centre for Research & Technology Hellas (CERTH), Thessaloniki, Greece; 3 Department of Organismal Biology, Evolutionary Biology Centre, Uppsala University, Uppsala, Sweden; 4 Naturalis Biodiversity Center, RA Leiden, The Netherlands; 5 The Natural History Museum, University of Oslo, Oslo, Norway; Chinese Academy of Medical Sciences, Peking Union Medical College, CHINA

## Abstract

Medicinal plants are used as a popular alternative to synthetic drugs, both in developed and developing countries. The economic importance of the herbal and natural supplement industry is increasing every year. As the herbal industry grows, consumer safety is one issue that cannot be overlooked. Herbal products in Thai local markets are commonly sold without packaging or labels. Plant powders are stored in large bags or boxes, and therefore buying local herbal products poses a high risk of acquiring counterfeited, substituted and/or adulterated products. Due to these issues, a reliable method to authenticate products is needed. Here DNA barcoding was used in combination with High Resolution Melting analysis (Bar-HRM) to authenticate three medicinal Acanthaceae species (*Acanthus ebracteatus*, *Andrographis paniculata* and *Rhinacanthus nasutus*) commonly used in Thailand. The *rbc*L barcode was selected for use in primers design for HRM analysis to produce standard melting profiles of the selected species. Melting data from the HRM assay using the designed *rbc*L primers showed that the three chosen species could be distinguished from each other. HRM curves of all fifteen test samples indicated that three of tested products did not contain the indicated species. Two closely related species (*A*. *paniculata* and *R*. *nasutus*), which have a high level of morphological similarity, were interchanged with one another in three tested products. Incorrect information on packaging and labels of the tested herbal products was the cause of the results shown here. Morphological similarity among the species of interest also hindered the collection process. The Bar-HRM method developed here proved useful in aiding in the identification and authentication of herbal species in processed samples. In the future, species authentication through Bar-HRM could be used to promote consumer trust, as well as raising the quality of herbal products.

## Introduction

### Herbal medicines

Natural products from plants have played a considerable role in the way of life of people around the world since ancient times. Plant products have been consumed as food and used as medicinal remedies. An enormous number of scientific reports highlight the benefits of using medicinal plants and herbs as an alternative to modern synthetic drugs [1–4]. It is clear that medicinal plants are a popular alternative to synthetic drugs. According to the World Health Organization [5], over 70% of the world’s population in developing countries uses herbal products. The past decade has seen the rapid growth of the herbal supplement and remedies market in many countries. Global Industry Analysts Inc. [6] report that the sale of these products has increased each year since 2004, with annual sales reaching 5.6 billion US dollars in 2012. It is estimated that the annual global sales of herbal supplement and remedies will reach up to 107 billion US dollar by the year 2017. The sale of these products in the US has risen for nine consecutive years since 2004. The report also notes that the global demand for herbal medicines continues to increase despite the economic recession. In addition to the US and Europe, Asia-Pacific and Japan also make up important markets for the global herbal supplement trade.

Many medicinal plant products have now been commercialized throughout various markets, including via the internet. These products are commonly sold in processed or modified forms such as powders, dried material, tablets, capsules and tea bags, making it almost impossible to accurately identify the constituent species [7,8]. Because of this, consumer safety could be a concern. Misidentification of the constituent plants may lead to the inclusion of undesirable, unrelated species, with a potential health risk to the end users. Substitution of the product’s ingredients either intentionally or inadvertently can have negative effect on both consumers and producers. Herbal products are often perceived to be safe due to their natural origin. However, counterfeited, substituted and adulterated products can put consumers in danger [9–11]. Recent advances in molecular techniques have made it possible to detect substitution and adulteration in medicinal products, even in processed forms [12,13].

Herbal products found in Thai local markets are commonly sold without packaging or labels. Wholesale quantities of plant powders may be stored in large unlabeled bags or boxes, and as a result those who buy local herbal products have a high risk of attaining counterfeited, substituted and/or adulterated products. Regardless of the sellers’ intention, buyers will never know whether the herbal powder they are buying is made of the species they intended to buy. Because of these identification issues, any measures that may aide in the identification of local herbal products would be beneficial. Many species from the Acanthaceae family are considered in Thailand to have health benefits, and three species (*Acanthus ebracteatus* Vahl, *Andrographis paniculata* (Burm.f.) Nees, and *Rhinacanthus nasutus* (L.) Kurz) are now included on the Thai National List of Essentials Medicine (NLEM; Thailand). These three Acanthaceae species are commonly used in Thai household as remedies for various ailments and thus are regularly sold at local markets. There is a need to find an approach that could help with the quality control of these herbal products to ensure both the satisfaction and safety of consumers.

### DNA barcoding

DNA barcoding was developed about a decade ago, and relies on a short, standardized regions of the genome to identify plant and animal species [14,15]. The mitochondrial DNA (mtDNA) cytochrome c oxidase subunit I (COI) gene was first chosen to be amplified and used to classify and identify butterfly species in the order Lepidoptera [16]. The results from this study show that butterfly species could be identified with 100% accuracy by using this short DNA region. Since then, the rapid development of the method, along with an increased frequency of DNA barcode use in many fields that require species-level determination of organisms including animals, plants and microorganisms has proven the popularity of molecular barcoding [17–20]. The use of COI contributed to the discovery of a significant number of new animal species, including fish, birds, mammals, marine organisms and insects. More than 50,000 (30%) of the butterfly species in the order Lepidoptera have been investigated using DNA barcoding [16,21–23].

Although the use of DNA barcoding in animals is relatively widespread, similar use of the technique in the plant kingdom has not caught on as quickly [24,25]. COI is not suitable for plant identification because the locus in plant mtDNA has a low mutation rate, which results in too little variation to sufficiently discriminate among plant species [26]. Instead, chloroplast DNA (cpDNA) is more suitable for DNA barcoding in plants [24]. Several DNA regions in the chloroplast genome have shown sufficient variation to be useful for plant species identification. The CBOL Plant Working Group studied 907 plant species using a variety of gene and non-gene regions in the cpDNA [25]. As a result, the group proposed the use of two regions, *rbc*L and *mat*K, as DNA barcodes in plants, as they exhibit a promising efficiency in plant species discrimination [25]. As an alternative to cpDNA markers, the nuclear DNA internal transcribed spacer (ITS) region was recently also suggested as a good choice for species discrimination in plants [27–29].

Although DNA barcoding is proven useful for species-level identification of plants, there are some limitations to the technique. It is costly and time-consuming, and not easy to apply routinely in developing countries due to financial constraints and limited availability of perishable chemicals and consumables. The need to develop and validate a method that is still reliable, but more economical and rapid than DNA barcoding is an ongoing challenge. Here we applied DNA barcoding with high resolution melting (Bar-HRM) analysis for species identification and authentication. The use of Bar-HRM for taxonomic identification and the detection of adulteration in food and agriculture products has been reported recently [30–32]. In this study we evaluate whether the technique is equally useful for species discrimination in constituents of herbal products.

## Methods

### Primers used for HRM analysis

Sequences of the plastid DNA region, *rbc*L of selected medicinal plants from the family Acanthaceae were extracted from GenBank (at the end of February 2014) using the key phrases “the name of locus” and “the name of species” in the annotations. Generally, sequences obtained from public databases, including GenBank, are of low quality with no known associated herbarium vouchers. For this reason, all of the sequences were subjected to critical evaluation and any low-quality sequences were removed. After processing, multiple alignments were made from the selected sequences using MEGA6 [33] and variable characters were calculated for the design of primers to be used for high resolution melting (HRM) analysis. Two main criteria were considered in order to obtain successful results in the HRM analysis: (i) the primer pair should generate a PCR product not exceeding 300 bp, (ii) the primer pairs should cover enough variable sites to enable discrimination among the tested species.

### Plant materials and DNA isolation

Both fresh and dried samples were included in this study. Three commonly used Thai herb species (*Ac*. *ebracteatus*, *An*. *paniculata* and *R*. *nasutus*) were the main focus of the study. Fresh specimens of these species were collected from The Garden of Medicinal Plants at the Faculty of Pharmacy, Chiang Mai. Dried plant tissues for DNA extraction were kindly provided by Queen Sirikit Botanic Garden (QSBG) from the following herbarium vouchers (*Ac*. *ebracteatus*: QSBG voucher no. 29333, *An*. *paniculata*: QSBG voucher no. 68296 and *R*. *nasutus* QSBG voucher no. 63282). The plant material was ground with liquid nitrogen, and 100 mg of fine powder was then used for DNA extraction with the Nucleospin Plant II kit (Macherey-Nagel, Germany) following the manufacturer’s instruction. The DNA was stored at −20° C for further use.

### High resolution melting (HRM) analysis

To determine the characteristic melting temperature (T_m_) for each sample that could be used to distinguish among the three different medicinal plants, DNA amplification using real-time PCR and DNA was performed using the Eco Real-Time PCR system (Illumina, San Diego, USA). The reaction mixture for the real-time PCR and HRM analysis consisted of a total volume of 10 μl, containing 5 μl of 2× THUNDERBIRD SYBR qPCR Mix, 0.2 μl of 10 mM forward primer, 0.2 μl of 10 mM reverse primer, 1 μl of 25 ng DNA and 3.6 μl of ddH_2_O. The primer pair was derived from the *rbc*L sequence data retrieved from an online database (Forward 5’-TAGACCTTTTTGAAGAAGGTTCTGT-3’ and Reverse 5’- TGAGGCGGRCCTTGGAAAGTT-3’). SYBR fluorescence dye was used to monitor both the accumulation of the amplified product and the high-resolution melting process in order to derive the T_m_ value during PCR.

The thermocycling reactions (PCR) were conducted in a 48-well Helixis plate using an initial denaturing step of 95°C for 5 min followed by 35 cycles of 95°C for 30 s, 57°C for 30 s, and 72°C for 20 s. The fluorescent data were acquired at the end of each extension step during the PCR. Before HRM, the products were denatured at 95°C for 15 s, and then annealed at 50°C for 15 s to randomly form DNA duplexes. For the HRM experiments, fluorescence data were collected every 0.1°C. Eco software (version 4.0.7.0) was used to analyze the T_m_. The negative derivative of the fluorescence (F) over temperature (T) (dF/dT) curve displays the T_m_, and the normalized raw curve depicts the decreasing fluorescence vs. increasing temperature. To generate normalized melting curves and differential melting curves [34], pre- and post-melt normalization regions were set to define the temperature boundaries of the normalized and difference plots that were used. *An*. *paniculata* was set as the reference species.

### Authenticating test of herbal products sold on Thai local markets

Fifteen local products were purchased for this study. All of the products were acquired in powder form, without labeling and/or proper packaging. According to the sellers, two of the products were comprised of *Ac*. *ebracteatus*, eight products were comprised of *An*. *paniculata* and the remaining five were comprised of *R*. *nasutus* (Table 1). Total DNA was extracted from each sample and then used in HRM analysis in order to identify the characteristic melting temperature (T_m_).

**Table 1:** Bar-HRM identifications of the tested products.

Product number	Point of purchase	Latitude, Longitude coordinates	Putative species	Bar-HRM species identification
1	Ratchawong Road, Muang Chiang Mai	18.791346, 98.998703	*A*. *ebracteatus*	*A*. *ebracteatus*
2	Saturday Night Market, Sankamphaeng, Chiang Mai	18.742670, 99.122488	*A*. *ebracteatus*	*A*. *ebracteatus*
3	Warorot Market, Muang, Chiang Mai	18.790688, 99.001001	*R*. *nasutus*	*R*. *nasutus*
4	Ratchawong Road, Muang Chiang Mai	18.791346, 98.998703	*R*. *nasutus*	*R*. *nasutus*
5	Nawarat Market, Muang, Chiang Mai	18.791159, 99.000165	*R*. *nasutus*	*R*. *nasutus*
6	Warorot Market, Muang, Chiang Mai	18.790718, 99.000979	*R*. *nasutus*	*A*. *paniculata* *
7	Saturday Night Market, Sankamphaeng, Chiang Mai	18.742670, 99.122488	*R*. *nasutus*	*R*. *nasutus*
8	Ratchawong Road, Muang Chiang Mai	18.791346, 98.998703	*A*. *paniculata*	*A*. *paniculata*
9	Nawarat Market, Muang, Chiang Mai	18.791159, 99.000165	*A*. *paniculata*	*A*. *paniculata*
10	Tonlamyai market, Muang, Chiang Mai	18.790287, 99.001289	*A*. *paniculata*	*A*. *paniculata*
11	Warorot Market, Muang, Chiang Mai	18.790688, 99.001001	*A*. *paniculata*	*R*. *nasutus* *
12	Thapae Road, Muang, Chiang Mai	18.788176, 98.996893	*A*. *paniculata*	*A*. *paniculata*
13	Sunday Night Market Walking Street, Tha Pae Gate, Chiang Mai	18.787761, 98.993224	*A*. *paniculata*	*A*. *paniculata*
14	Saturday Night Market Walking Street, Wua Lai Road, Chiang Mai	18.778231, 98.984167	*A*. *paniculata*	*R*. *nasutus* *
15	Saturday Night Market, Sankamphaeng, Chiang Mai	18.742670, 99.122488	*A*. *paniculata*	*A*. *paniculata*

*Species found to be different from the species indicated by the seller

## Results

### Data mining and primers used

**Fig 1:**
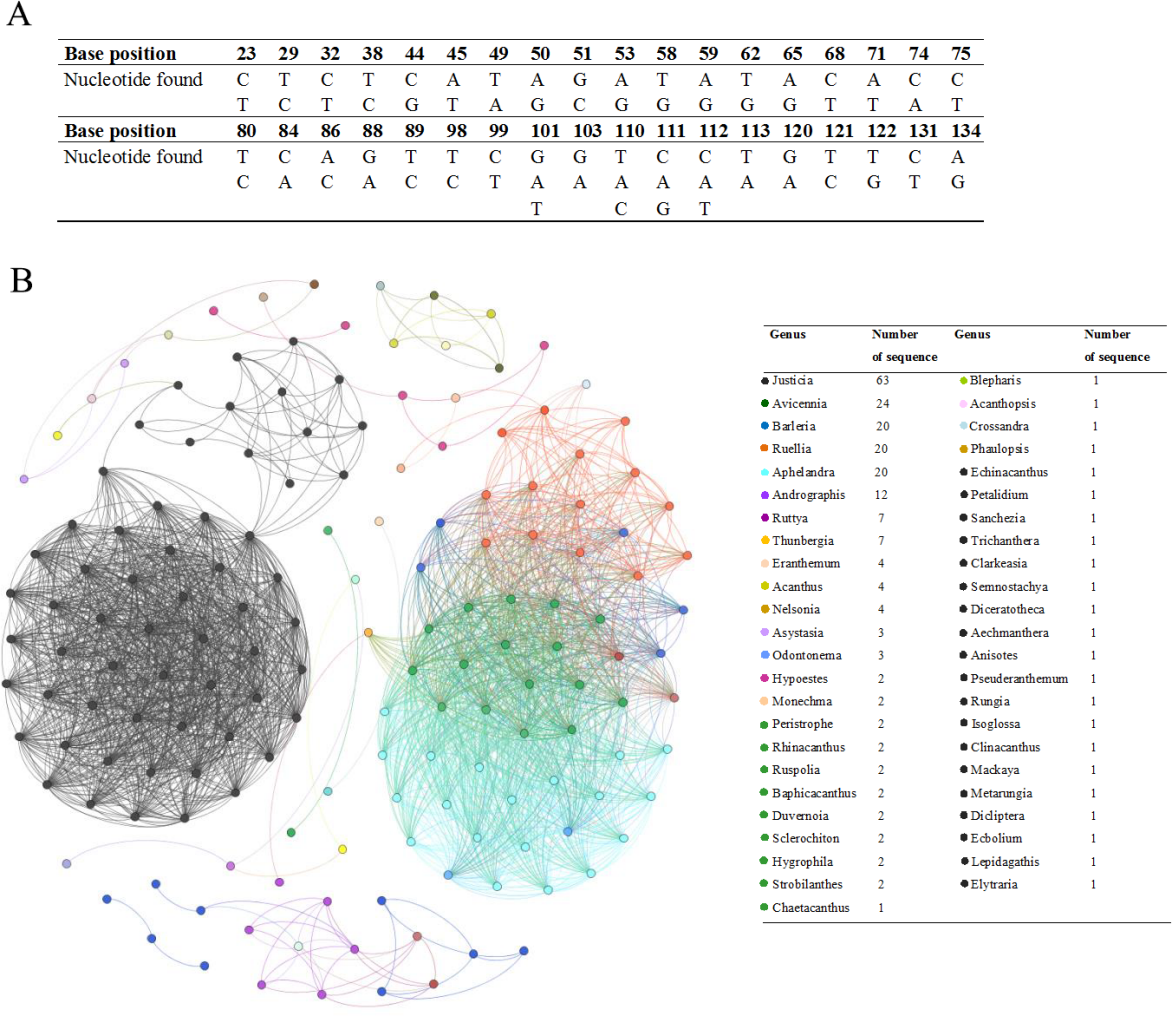
Variable sites and Distance plot of the sequences retrieved from GenBank. A) Nucleotide variation in the 136 bp *rbc*L fragment of 235 aligned Acanthaceae species. B) Gelphi distance plot of the *rbc*L sequences belonging to 47 genera of Acanthaceae downloaded from GenBank.

The amplification of the *rbc*L locus from three medicinal plant species in this study was performed using specific primers corresponding to the *rbc*L barcode region. All *rbc*L sequences of Acanthaceae were extracted from GenBank and the variable characters, average distance, and average %GC content were calculated for all samples using MEGA6. In total, 248 sequences were retrieved, of which 235 sequences were deemed useful for further analysis (S1 Table). An alignment of all useful sequences was made, and a 136 nucleotide base-pair fragment was analyzed after its amplification with newly developed forward and reverse primers. Thirty-six variable sites (26.5%) and 41.2% GC content were observed within the fragment (Fig 1A). The average distance was calculated for each sequence and plotted using Gephi [35] (Fig 1B). Fig 1B shows that all sequences from samples of the same genus cluster together. The majority of *rbc*L sequences are from *Justicia*. Commonly used Thai herbs belong predominantly to *Andrographis*, *Acanthus* and *Thunbergia*, though sequences from these genera are limited in our reference dataset. Among the total of 235 analyzed sequences, we found only one mismatched nucleotide position on the reverse primer site, and a perfect match among all samples for the forward primer. Thus, these newly developed primers, which were designed based on the sequences extracted from GenBank, were predicted to perform well in HRM analyses with the medicinal plant species in question.

### HRM analysis

**Fig 2:**
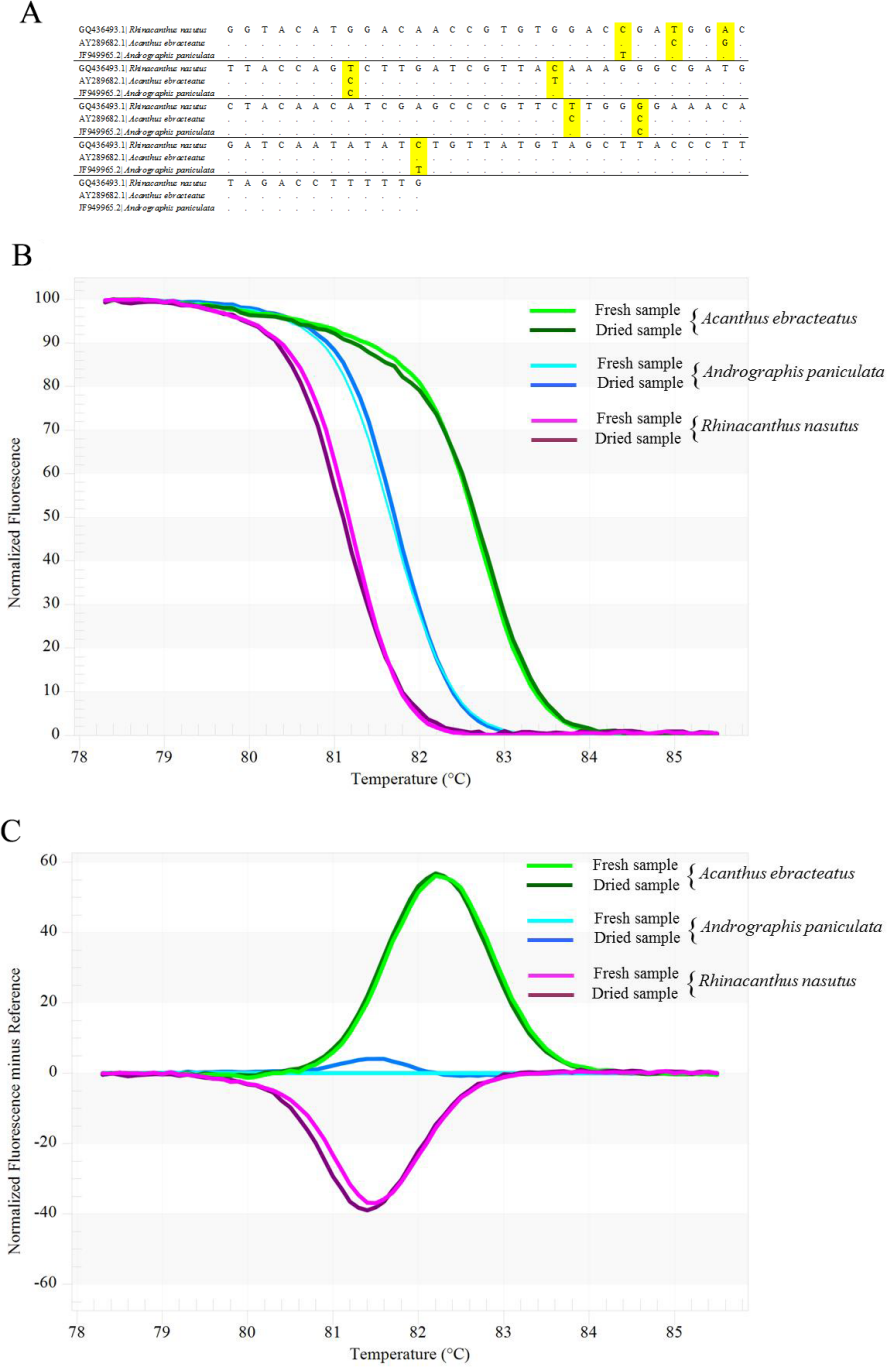
*rbc*L nucleotide alignment and melting profiles of the three Acanthaceae species. A) Variable sites detected in *rbc*L sequences of the three closely related species, *Acanthus ebracteatus*, *Andrographis paniculata* and *Rhinacanthus nasatus*. B) Normalized fluorescence plot of barcoding coupled high resolution melting (Bar-HRM). C) Reference-corrected normalized fluorescence plot of barcoding coupled high resolution melting (Bar-HRM).

The newly designed *rbc*L primer pair yielded amplicons of the expected size, approximately 136 base-pairs long. Eight variable sites were observed within the fragment of the three tested herb species (Fig 2A). The amplicons were analyzed using HRM to determine the T_m_. Fig 2B and 2C present the analysis by means of conventional derivative plots, which show that the T_m_ value for the *rbc*L fragment from each species was represented by a peak. The melt curve is generated by slowly melting the DNA of tested plant species through a range of temperatures in the presence of a dsDNA binding dye. The melting temperature peaks of the tested plant species are calculated as T_m_. HRM analysis with this primer pair proved to be a powerful tool for the identification of the three closely related Acanthaceae species (*Ac*. *ebracteatus*, *An*. *paniculata* and *R*. *nasutus*). The individual melting curves were reproducibly achieved from each of the three different species in triplicate analyses. Similar melting curves were achieved from the same species regardless of whether the DNA template was extracted from fresh or dried tissue (Fig 2B and 2C). Thus, the method may also be applicable for processed herbal products that still contain tissue material with intact DNA.

### Identification of herbal species in local products

**Fig 3:**
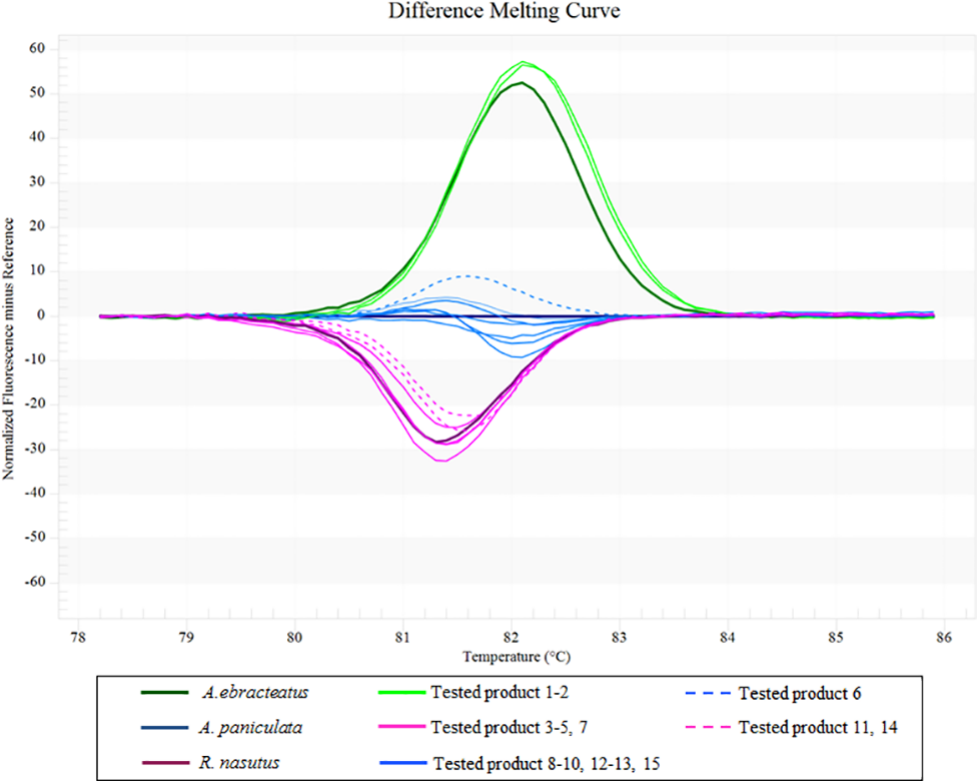
Representative profiles of the melting curves (difference plot curves). Bar-HRM difference plot curves obtained using the *rbc*L primer pair to identify 15 local herbal products. Three tested products (6, 11 and 14) were found to be different species from the species indicated by the vendor.

Constituent species in herbal products bought from local markets in Thailand were investigated to assess the reliability of information regarding their ingredients, as the herbal products are often sold without proper packaging or labeling. Fifteen herbal products were purchased from local producers. The HRM analysis using *rbc*L primers was then performed to identify the species in the products and to see whether they are consistent with the species indicated by the sellers. The results of the analysis reveal that two samples which sellers identified as *An*. *paniculata* were actually *R*. *nasutus* (products no. 11 and 14), while one which sellers identified as *R*. *nasutus* was actually *An*. *paniculata* (product no. 6) (Fig 3).

## Discussion

Whether intentional or not, substitution of species is not something that should happen. It is not possible to tell whether a product is the indicated species based on visual inspection, as they are sold in powdered form. Moreover, other on-site methods of identifying the component species studied here may be ineffective as both species taste similar (bitter) and lack a distinctive smell. In fact, when the morphology of these two herbal species is examined it becomes evident that they are relatively similar as it may be hard for a non-expert to distinguish or identify them. The Bar-HRM method developed here seems to be an effective way to accurately identify species in herbal products. Although twelve out of fifteen (80%) products tested were identified as the same species as indicated by the seller (Fig 3), a higher sample number as well as the inclusion of samples from more markets could lead to lower figures than 80%. It is not surprising that the misidentification of species in local products is reported here. The two main issues involved in this misidentification are that i) there is no reliable approach for controlling or authenticating raw materials collected by local producers and ii) there is no regulation on proper packaging or labeling of herbal products sold in Thai markets.

Detecting substitution and adulteration in herbal medicines using molecular techniques has been done before and proven to be a successful method [8,12,13]. For instance, one study found that a product labeled as black cohosh (*Actaea racemosa* L.), one of the top ten most popular herbal supplements sold in the US, actually contained three related Asian *Actaea* species that can be toxic to humans [36]. Studies in Morocco of the medicinal herbs trade found widespread substitutions of products sold on the markets [8,11,37]. It also showed that identifications made by herbalists selling retail herbal medicines were often inaccurate [37]. Another recent study of herbal medicines in North America that included 44 herbal products showed that almost half of the products contained species that were not listed among the main ingredients on the label [13]. Surprisingly, another third of the products tested contained none of the ingredients indicated on the label [13]. Several other studies have raised similar concerns from their findings. A study of 146 commercial herbal teas based on either *rbc*L or *mat*K barcodes revealed that 35% of the samples were contaminated with ingredients that were not on the labels [38]. A study of Korean and American ginseng using *rbc*L, *mat*K and ITS barcodes found that 50% of the ginseng products examined in the study contained American ginseng (*Panax quinquefolius* L.) instead of Asian ginseng (*Panax ginseng* C.A.Mey.) [39].

These examples have all used standard Sanger barcoding or metabarcoding approaches, but the use of Bar-HRM to study herbal medicine substitution has not been reported before [40]. Bar-HRM of botanical products has been successfully used for the authentication of an EU Protected Designation of Origin product made from *Lathyrus clymenum* [41], for olive oil and adulterants [42], for species distinction in Mediterranean pines [31], for detection of allergenic hazelnut contamination [32], for processed bean crops [30,43,44]. Bar-HRM is quickly gaining popularity in application due to its low direct and indirect costs and high accuracy of detection.

## Conclusion

Pharmacovigilance of herbal medicines relies on product label information of ingredients and the adherence to good manufacturing practices along the commercialization chain. Several studies have shown that substitution of plant species occurs in herbal medicines, and this in turn poses a challenge to herbal pharmacovigilance as adverse reactions might be due to substituted ingredients. Bar-HRM analysis has been proven to be a fast and reliable technique for the authentication of herbal products. Here, we describe the development of a Bar-HRM method that can be used to test for the adulteration of commonly used herbal products. The tested products were traded as processed powder, which impedes conventional identification. Because of this processing it is almost impossible to identify which herbal species are present in products using morphological characters. The DNA extracted from all products tested yielded a specific amplification product with the designed *rbc*L Bar-HRM primers. The normalised HRM curves for the amplicons, from the three species (*An*. *paniculata*, *Ac*. *ebracteatus* and *R*. *nasutus*) and 15 herbal products, based on HRM analysis with barcode marker *rbc*L were easily distinguished, and 12 of the 15 tested samples were successfully assigned to the species indicated by the seller. However, three products were found to contain plant species that were different from the species indicated by the seller. Therefore, the developed method could be easily used for rapid and low-cost authentication of herbal products.

## Supporting Information

S1 Table Acanthaceae sequences of rbcL were retrieved from GenBank (NCBI) for each of the genus with accession number. (DOCX)
