# Supplementary material for: Bar-HRM for Authentication of Plant-Based Medicines: Evaluation of Three Medicinal Products Derived from Acanthaceae Species
Source: PLoS One. 2015 May 26;10(5):e0128476. doi: 10.1371/journal.pone.0128476 (PMC4444109; doi:10.1371/journal.pone.0128476)
Supplement: S1 Table — (DOCX) [file pone.0128476.s001.docx]

**S1 Table.** **Acanthaceae sequences of *rbcL* were retrieved from GenBank (NCBI) for each of the genus with accession number.**

| **Genus** | **Sequence** | **Acession number (NCBI)** | **Genus** | **Sequence** | **Acession number (NCBI)** |
| --- | --- | --- | --- | --- | --- |
| *Acanthus* | 4 | AY289682, L12592 | *Ecbolium* | 1 | JQ933315 |
|  |  | HE963302, HM849737 | *Echinacanthus* | 1 | JQ933316 |
| *Aechmanthera* | 1 | JQ933209 | *Elytraria* | 1 | AF188127 |
| *Andrographis* | 9 | GQ436494-GQ436496 | *Eranthemum* | 4 | JQ734505-JQ734506 |
|  |  | JQ230990, JQ922118 |  |  | JQ933327, JQ933456 |
|  |  | JQ933217, JF949965 | *Hypoestes* | 3 | AB586152-AB586153 |
|  |  | KF365996, KF425766 |  |  | L12593 |
| *Anisotes* | 1 | JF265288 | *Hygrophila* | 2 | GU135241, GU135244 |
| *Aphelandra* | 20 | GQ981668, L01884 | *Isoglossa* | 1 | AM234780 |
|  |  | JQ590007-JQ590024 | *Justicia* | 62 | JQ590033-JQ590068 |
| *Asystasia* | 3 | GU135172, JQ933229 |  |  | JQ231000, JX572702 |
|  |  | JQ673521 |  |  | JQ594958-JQ594960 |
| *Avicennia* | 22 | AY008829-AY008832 |  |  | KC756924-KC756934 |
|  |  | AY289681, JX572318 |  |  | KF669388-KF669393 |
|  |  | JQ590025-JQ590030 |  |  | DQ006045, L01930 |
|  |  | JQ594360-JQ594364 |  |  | GQ436497, GQ436500 |
|  |  | JQ594977-JQ594981 |  |  | HM850082, L14401 |
|  |  | U28868 | *Lepidagathis* | 1 | L12594 |
| *Baphicacanthus* | 2 | GQ436498-GQ436499 | *Mackaya* | 1 | JX572742 |
| *Barleria* | 16 | L01886, JQ673524 | *Metarungia* | 1 | JF265518 |
|  |  | AB586149-AB586151 | *Monechma* | 2 | AM234781, AB586154 |
|  |  | JF265299-JF265300 | *Nelsonia* | 4 | HQ384879, L01935 |
|  |  | JQ590031-JQ590032 |  |  | JQ590069-JQ590070 |
|  |  | KF890169-KF890172 | *Odontonema* | 3 | JQ590071-JQ590073 |
|  |  | JQ231001 | *Peristrophe* | 2 | AM234782, KF425772 |
| *Blepharis* | 1 | JQ673527 | *Petalidium* | 1 | JQ933440 |
| *Chaetacanthus* | 1 | AM234779 | *Phaulopsis* | 1 | JQ933444 |
| *Clarkeasia* | 1 | JQ933269 | *Ruellia* | 20 | GU135168, GU135171 |
| *Clinacanthus* | 1 | GQ436501 |  |  | GU135266, L12595 |
| *Crossandra* | 1 | JQ933287 |  |  | AB586155-AB586156 |
| *Diceratotheca* | 1 | JX469440 |  |  | JQ673547-JQ673548 |
| *Dicliptera* | 1 | JQ933303 |  |  | JQ590074-JQ590085 |
| *Duvernoia* | 2 | JF265402-JF265403 | *Rhinacanthus* | 2 | KF381120, GQ436493 |

**S1 Table. (Continued) Acanthaceae sequences of *rbcL* were retrieved from GenBank (NCBI) for each of the genus with accession number.**

| **Genus** | **Sequence** | **Acession number (NCBI)** |
| --- | --- | --- |
| *Rungia* | 1 | JQ933466 |
| *Ruspolia* | 2 | JF265577 |
|  |  | JX572942 |
| *Ruttya* | 7 | AB586157-AB586161 |
|  |  | JF265578 |
|  |  | L02434 |
| *Sanchezia* | 1 | AJ247613 |
| *Sclerochiton* | 2 | JX572957-JX572958 |
| *Strobilanthes* | 2 | JQ933455 |
|  |  | JQ933492 |
| *Thunbergia* | 6 | AM234783 |
|  |  | AY008828 |
|  |  | L12596 |
|  |  | HQ384878 |
|  |  | JQ590086 |
|  |  | KF181493 |
| *Trichanthera* | 1 | GQ981903 |
